# Age and efficacy of remote ischemic conditioning in acute ischemic stroke

**DOI:** 10.1111/cns.14451

**Published:** 2023-09-04

**Authors:** Yu Cui, Jing Zhang, Hui‐Sheng Chen

**Affiliations:** ^1^ Department of Neurology General Hospital of Northern Theater Command Shenyang China; ^2^ Yinchuan Dingxiang Internet Hospital Yinchuan China

**Keywords:** acute ischemic stroke, age, functional outcome, remote ischemic conditioning

## Abstract

**Aims:**

A post hoc analysis of RICAMIS trial to evaluate functional outcomes in relation to patient age.

**Methods:**

Patients in RICAMIS were divided into six age groups. The primary outcome was excellent functional outcome at 90 days, defined as modified Rankin Scale (mRS) score of 0–1. Compared with patients receiving usual care alone, we investigated the association of remote ischemic conditioning (RIC) effect with functional outcomes in each group and the interaction between RIC effect and age.

**Results:**

Of 1776 patients, 498 were assigned to <60 years, 326 to 60 to <65 years, 325 to 65 to <70 years, 278 to 70 to <75 years, 206 to 75 to <80 years, and 143 to ≥80 years. Higher proportions of primary outcome were found associated with RIC in <60 years group (72.6% vs. 64.8%; adjusted risk difference [RD], 6.8%; 95% CI, −1.6% to 15.1%; *p* = 0.11), 60 to <65 years group (70.7% vs. 67.1%; adjusted RD, 3.1%; 95% CI, −7.2% to 13.3%; *p* = 0.56), 65 to <70 years group (70.5% vs. 63.6%; adjusted RD, 3.5%; 95% CI, −6.8% to 13.8%; *p* = 0.51), 70 to <75 years group (59.7% vs. 54.9%; adjusted RD, 4.7%; 95% CI, −7.1% to 16.4%; *p* = 0.61), 75 to <80 years group (61.5% vs. 55.9%; adjusted RD, 5.7%; 95% CI, −7.8% to 19.1%; *p* = 0.41), and ≥ 80 years group (59.2% vs. 59.7%; adjusted RD, −2.6%; 95% CI, −18.8% to 13.5%; *p* = 0.75). No significant interaction between RIC effect and age was found among groups.

**Conclusions:**

This is the first report that RIC effect may be attenuated with increasing age in patients with acute moderate ischemic stroke with respect to functional outcome.

## Graphical abstract

Remote ischemic postconditioning may be effective across age with respect to excellent functional outcome at 90 days, but the effect will be attenuated with increasing age in patients with acute moderate ischemic stroke who are not candidate for intravenous thrombolysis or endovascular therapy.

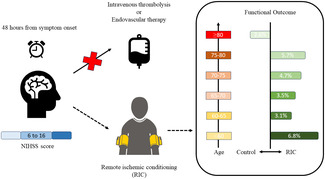

## 1 INTRODUCTION

Based on data from clinical trials,^1^, ^2^ current guideline recommends reperfusion therapies as standard treatments for acute ischemic stroke,^3^ which include intravenous thrombolysis and endovascular thrombectomy. However, the use of reperfusion therapy is limited by strict eligibility criteria and imperfect treatment effects.^3^, ^4^ It has been a hot topic to explore cerebral protection as an adjunct therapy to improve the prognosis of stroke for decades.^5^ Up to date, few cerebral protective strategies have been translated from preclinical to clinical practice.^6^

Remote Ischemic Conditioning (RIC), intermittently blocking the blood flow of limbs and producing transient ischemic with the intention of protecting brain, has been demonstrated to improve neurologic function in both experimental stroke models and clinical trial.^7^, ^8^ Recently, the Remote Ischemic Conditioning for Acute Moderate Ischemic Stroke (RICAMIS) trial demonstrated that RIC, which was conducted twice a day for 10–14 days and initiated within 48 h of stroke onset, safely and significantly improved excellent functional outcome at 90 days among patients with acute moderate ischemic stroke who did not receive any reperfusion therapy.^9^ In RICAMIS and another recent randomized clinical trial of RIC treatment for ischemic stroke with large sample size,^9^, ^10^ the prespecified subgroup analysis for primary outcome showed that older patients (age ≥65 years) have less efficacy of RIC treatment than those younger (age <65 years). Furthermore, accumulating evidence shows that patient age is an important predictor of functional outcome after stroke,^11^, ^12^ and increased age is often closely linked with poor function recovery.^13^, ^14^ However, there is no study investigating the effect of patient age on RIC treatment for functional outcome after stroke so far.

The data from the RICAMIS trial provide the first opportunity to further examine the effect of age on the clinical outcomes after RIC treatment in patients with acute ischemic stroke who are not candidate for intravenous thrombolysis or endovascular therapy, as patients with a wide range of age and without any reperfusion therapy were included in the large‐sample trial.

## 2 METHODS

### 2.1 Study design and participants

Details on the design, protocol, and statistical analysis plan of RICAMIS have been published.^9^, ^15^ In brief, RICAMIS trial was a multicenter, open‐label, blinded‐endpoint, randomized clinical trial enrolling 1893 patients between December 26, 2018, and April 19, 2021, to assess the efficacy of RIC treatment in patients with acute moderate ischemic stroke. Eligible patients were 18 years and older, had been functioning independently before stroke (modified Rankin Scale [mRS] scores, 0–1; range, 0 [no symptoms] to 6 [death]), and diagnosed with acute moderate ischemic stroke (National Institute Health of Stroke Scale [NIHSS] scores at admission, 6–16) within 48 h after stroke onset. Exclusion criteria were patients who received intravenous thrombolysis or endovascular therapy, had any contraindication for RIC treatment, or had cardiogenic embolism. The study was approved by the ethics committee of General Hospital of Northern Theatre Command (ethics approval ID: k2018[43]) and each participating center. All patients or their legally authorized representatives provided written informed consent before enrollment. The study was registered with ClinicalTrials.gov, number NCT03740971. Patients in the full analysis set of RICAMIS trial were included in this post hoc analysis.

### 2.2 Procedures

The effect of RIC on functional outcome was investigated within narrow age range in 5‐year age groups.^16^ According to patient age, eligible patients were divided into six groups: <60 years, 60 to <65 years, 65 to <70 years, 70 to <75 years, 75 to <80 years, and ≥80 years. In each group, patients were assigned into RIC group and control group according to whether they received RIC treatment as an adjunct to usual care based on current guideline.^3^ RIC treatment was performed by 5 cycles of cuff inflation (200 mm Hg for 5 min) and deflation (for 5 min), for a total procedure time of 50 min, twice daily for 10–14 days. Further detail of RIC treatment has been described in a previous report.^9^ Neurological status, measured with NIHSS score, was evaluated at admission. Follow‐up data including assessment of prognosis were collected at 90 days after randomization.

### 2.3 Outcomes

In this post hoc analysis of RICAMIS trial, the outcomes included long‐term functional outcomes in the primary study.^9^ The primary outcome was excellent functional outcome at 90 days, defined as a mRS score of 0–1. The secondary outcomes were favorable functional outcome at 90 days, defined as a mRS score of 0 to 2, and a shift in measure of function according to distribution on the ordinal mRS score at 90 days. The assessment of mRS score at 90 days was assessed in person or by telephone through blinded measurements by trained and certified assessors in each center who were unaware of treatment allocation or clinical details.^15^

### 2.4 Statistical analysis

The post hoc analysis was performed by intention‐to‐treat principle based on all randomized patients with at least one post‐baseline efficacy evaluation (full analysis set population). Data distribution was tested for normality by QQ‐plots. For baseline characteristics of eligible patients, we summarized continuous variables as median (interquartile range [IQR]) and categorical variables as frequencies (percentages). For treatment effect of outcomes, such as excellent functional outcome and favorable functional outcome at 90 days, we estimated absolute number of events, and absolute difference (risk difference [RD]) with their 95% confidence intervals (CIs). For treatment effect of mRS score distribution at 90 days, we estimated odds ratio (OR) with 95% CI.

First, probability of excellent functional outcomes at 90 days was respectively calculated in patients receiving RIC treatment as an adjunct to usual care and only usual care through binary logistic regression analysis, which included sex, premorbid function, NIHSS score at randomization, history of stroke or transient ischemic stroke, and time from onset of symptom to treatment as covariates. The probability curves with their 95% CIs stratified according to treatments were drawn.

Second, we compared the efficacy of RIC treatment with usual care in each age group. The primary analysis of this post hoc analysis was adjusted analysis accounting for baseline variables, which showed difference between groups with *p* value <0.1. Generalized linear models were performed to evaluate the association between RIC treatment and outcomes, such as excellent functional outcomes and favorable functional outcome at 90 days. The generalized linear model has a binomial distribution, and identity link functions, which will generate RD of having outcomes between RIC and control groups together with two‐sided 95% CIs and *p* values. Ordinal logistic analysis was performed to evaluate the association between RIC treatment and mRS distribution at 90 days. To avoid non‐convergence when all covariates were introduced into the adjusted analyses simultaneously, we calculated a propensity score with treatment as the dependent variable and baseline covariates with difference (*p* value <0.1) as independent variables through a logistic regression model and then included the calculated propensity score (continuous variable) as a covariate in the model for adjusted analysis. Missing data of covariates included in the adjusted analyses were imputed through simple imputation. Briefly, missing values for continuous variables were imputed from random values assuming a normal distribution with mean and standard deviation calculated from the available sample, and missing values for count variables were imputed from random values from a Poisson distribution with mean *λ* estimated from the available sample. Unadjusted analyses for primary and secondary outcomes were also performed in the population as sensitive analyses.

Third, the assessments of association between patient age and effect of RIC treatment on primary and secondary outcomes were conducted by generalized linear model or ordinal regression analysis with the treatment, age groups, and their interaction term as independent variables, and the *p* value presented for the interaction term. The adjusted interactions were conducted through including imbalance baseline variables between <60 years and each other age groups with *p* value <0.1 in the above models. The adjusted analyses were performed with similar methods of propensity score used in the analysis of association between RIC treatment and outcomes.

Fourth, to address small sample size in each age group, we made a sensitivity analysis for primary outcome by dividing patients into three age group (< 60 years, 60 to <70 years, and ≥70 years).

All analyses presented were exploratory, and all *p* values were nominal. Two‐sided *p* values <0.05 were considered significant. All statistical analyses were performed using the SPSS software (version 26.0, IBM) and R software (version 4.1.0, R Foundation for Statistical Computing).

## 3 RESULTS

**FIGURE 1:**
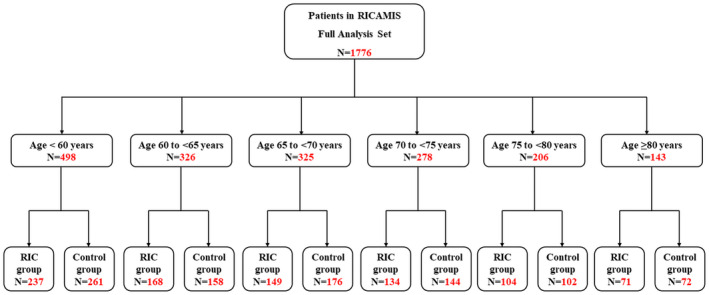
Study Flowchart. Age was limited with equal to or more than 18 years in the RICAMIS trial and finally ranged from 25 to 93 in full analysis set population. Control group included patients who received no RIC treatment. RIC, remote ischemic conditioning; RICAMIS, Remote Ischemic Conditioning for Acute Moderate Ischemic Stroke.

A total of 1776 patients from full analysis set of RICAMIS trial were included in this post hoc analysis, including 498 (237 in RIC group and 261 in Control group) in the age <60 years, 326 (168 in RIC group and 158 in Control group) in the age 60 to <65 years, 325 (149 in RIC group and 176 in Control group) in the age 65 to <70 years, 278 (134 in RIC group and 144 in Control group) in the age 70 to <75 years, 206 (104 in RIC group and 102 in Control group) in the age 75 to <80 years, and 143 (71 in RIC group and 72 in Control group) in the age ≥80 years (Figure 1). The median (IQR) age was 65 (58–73) years, and 606 (34.1%) patients were women. Details of baseline clinical characteristics among age groups are shown in Table 1, and those among treatment groups of each age group are shown in Table 2. The imbalanced baseline characteristics with *p* value <0.1 were adjusted by propensity score in the primary analysis.

**TABLE 1:** Baseline characteristics of patients between age groups.

	<60 years (*N* = 498)	60 to <65 years (*N* = 326)	65 to <70 years (*N* = 325)	70 to <75 years (*N* = 278)	75 to <80 years (*N* = 206)	≥80 years (*N* = 143)	*p* Value
Age, y	54 (50–57)	62 (61–63)	67 (66–68)	72 (71–73)	77 (76–78)	83 (81–85)	<0.001*
Sex (F)	116 (23.3)	99 (30.4)	109 (33.5)	113 (40.6)	103 (50.0)	66 (46.2)	<0.001*
Current smoker	202/484 (41.7)	118/312 (37.8)	86/314 (27.4)	47/273 (17.2)	32/194 (16.5)	20/140 (14.3)	<0.001*
Current drinker^a^	102/488 (20.9)	48/314 (15.3)	37/319 (11.6)	28/274 (10.2)	19/201 (9.5)	6/139 (4.3)	<0.001*
Hypertension	301/492 (61.2)	190/320 (59.4)	216/323 (66.9)	167/275 (60.7)	121/202 (59.9)	88/141 (62.4)	0.43
Diabetes	124/497 (24.9)	83/325 (25.5)	93/324 (28.7)	66/276 (23.9)	39/205 (19.0)	26 (18.2)	0.08
Previous ischemic or hemorrhagic stroke^b^	120 (24.1)	112/324 (34.6)	116/321 (36.1)	106/276 (38.4)	71/204 (34.8)	44/142 (31.0)	<0.001*
Previous transient ischemic attack	6 (1.2)	7/325 (2.2)	3/323 (0.9)	3/277 (1.1)	2 (1.0)	1 (0.7)	0.70
Systolic blood pressure, mmHg	150 (140–163)	150 (140–165)	150 (140–165)	150 (140–164)	157 (140–168)	150 (137–169)	0.42
Diastolic blood pressure, mmHg	90 (82–100)	90 (80–98)	90 (80–96)	90 (80–96)	86 (79–94)	84 (78–90)	<0.001*
Fasting blood glucose at randomization, mmol/L	6.29 (5.36–8.91)	6.23 (5.32–8.31)	6.42 (5.51–8.55)	6.40 (5.40–7.98)	6.27 (5.40–8.10)	6.01 (5.36–7.44)	0.21
NIHSS score at randomization^c^	7 (6–9)	7 (6–9)	7 (6–8)	7 (6–9)	7 (6–10)	7 (6–10)	0.27
Onset‐to‐treatment time, hour	26.2 (13.0–37.0)	24.9 (11.8–34.7)	27.1 (15.0–35.3)	25.3 (14.8–34.5)	21.6 (10.7–31.0)	24.1 (12.0–33.1)	0.02*
Estimated premorbid function (mRS)^d^							
No symptoms (score, 0)	380 (76.3)	241 (73.9)	246 (75.7)	209 (75.2)	155 (75.2)	101 (70.6)	0.82
Symptoms without any disability (score, 1)	118 (23.7)	85 (26.1)	79 (24.3)	69 (24.8)	51 (24.8)	42 (29.4)	
Presumed stroke cause^e^							
Undetermined cause	257/497 (51.7)	180/325 (55.4)	163 (50.2)	151/277 (54.5)	98 (47.6)	80 (55.9)	0.63
Large artery atherosclerosis	132/497 (26.6)	91/325 (28.0)	102 (31.4)	83/277 (30.0)	69 (33.5)	39 (27.3)	
Small artery occlusion	93/497 (18.7)	47/325 (14.5)	55 (16.9)	37/277 (13.4)	32 (15.5)	20 (14.0)	
Other determined cause	9/497 (1.8)	5/325 (1.5)	1 (0.3)	3/277 (1.1)	3 (1.5)	1 (0.7)	
Cardioembolic	6/497 (1.2)	2/325 (0.6)	4 (1.2)	3/277 (1.1)	4 (1.9)	3 (2.1)	
Duration of hospitalization, day	11 (10–12)	11 (10–12)	11 (10–12)	11 (10–12)	10 (9–12)	11 (9–12)	0.12

*Note*: Data were shown with number (percentage, %) or median (interquartile range). *p* value showed the difference of baseline characteristics compared between groups.

Abbreviations: mRS, modified Rankin Scale; NIHSS, National Institute of Health Stroke Scale; RIC, remote ischemic conditioning.

* *p* value <0.05.

^a^ Current drinker means consuming alcohol at least once a week within 1 year before the onset of the disease and consuming alcohol continuously for more than 1 year.

^b^ Previous ischemic stroke referred only to the patients with pre‐stroke mRS ≤1.

^c^ Patients with NIHSS scores of 6 to 16 were eligible for this study; NIHSS scores range from 0 to 42, with higher scores indicating more severe neurologic deficit.

^d^ Scores on the modified Rankin Scale (mRS) of functional disability range from 0 (no symptoms) to 6 (death).

^e^ The presumed stroke cause was classified according to the Trial of Org 10,172 in Acute Stroke Treatment (TOAST) classification systemusing clinical findings, brain imaging, and laboratory tests. Other determined causes included pulmonary embolism, peripheral vessel incident, and cardiovascular incident.^17^

**TABLE 2:** Baseline Characteristics of Patients Between RIC and Control Groups According to Age Groups.

	<60 years			60 to <65 years			65 to <70 years		
	RIC (*N* = 237)	Control (*N* = 261)	*p* Value	RIC (*N* = 168)	Control (*N* = 158)	*p* Value	RIC (*N* = 149)	Control (*N* = 176)	*p* Value
Age, y	55 (50–57)	54 (50–56)	0.07	62 (61–63)	62 (61–63)	0.32	67 (66–68)	67 (66–68)	0.99
Sex (F)	51 (21.5)	65 (24.9)	0.37	53 (31.5)	46 (29.1)	0.63	55 (36.9)	54 (30.7)	0.24
Current smoker	104/231 (45.0)	98/253 (38.7)	0.03	66/164 (40.2)	52/148 (35.1)	0.21	38/142 (26.8)	48/172 (27.9)	0.86
Current drinker^a^	59/233 (25.3)	43/255 (16.9)	0.06	34/164 (20.7)	14/150 (9.3)	0.03	17/145 (11.7)	20/174 (11.5)	0.85
Hypertension	142/235 (60.4)	159/257 (61.9)	0.74	92/165 (55.8)	98/155 (63.2)	0.17	108/148 (73.0)	108/175 (61.7)	0.03^*^
Diabetes	61 (25.7)	63/260 (24.2)	0.70	42 (25.0)	41/157 (26.1)	0.82	44 (29.5)	49/175 (28.0)	0.76
Previous ischemic or hemorrhagic stroke^b^	56 (23.6)	64 (24.5)	0.82	59/167 (35.3)	53/157 (33.8)	0.77	57 (38.3)	59/172 (34.3)	0.46
Previous transient ischemic attack	3 (1.3)	3 (1.1)	0.91	3/167 (1.8)	4 (2.5)	0.65	2/148 (1.4)	1/175 (0.6)	0.47
Systolic blood pressure, mmHg	150 (140–164)	150 (140–163)	0.59	150 (140–160)	152 (140–165)	0.09	150 (140–168)	150 (140–163)	0.73
Diastolic blood pressure, mmHg	90 (84–100)	90 (80–100)	0.76	90 (80–98)	90 (80–99)	0.04	90 (80–96)	90 (80–97)	0.94
Fasting blood glucose at randomization, mmol/L	6.45 (5.40–8.91)	6.20 (5.30–8.95)	0.35	6.06 (5.24–8.30)	6.35 (5.40–8.46)	0.41	6.32 (5.55–9.13)	6.60 (5.45–8.31)	0.67
NIHSS score at randomization^c^	7 (6–9)	7 (6–9)	0.62	7 (6–9)	7 (6–8)	0.49	7 (6–8)	7 (6–9)	0.97
Onset‐to‐treatment time, hour	27.0 (12.6–38.0)	25.9 (13.9–35.2)	0.42	25.5 (11.8–35.7)	24.1 (12.1–34.1)	0.22	28.0 (17.1–36.8)	25.5 (13.3–34.6)	0.30
Estimated premorbid function (mRS)^d^									
No symptoms (score, 0)	184 (77.6)	196 (75.1)	0.51	120 (71.4)	121 (76.6)	0.29	114 (76.5)	132 (75.0)	0.75
Symptoms without any disability (score, 1)	53 (22.4)	65 (24.9)		48 (28.6)	37 (23.4)		35 (23.5)	44 (25.0)	
Presumed stroke cause^e^									
Undetermined cause	138 (58.2)	119/260 (45.8)	0.07	93 (55.4)	87/157 (55.4)	0.57	88 (59.1)	75 (42.6)	0.01^*^
Large artery atherosclerosis	57 (24.1)	75/260 (28.8)		43 (25.6)	48/157 (30.6)		40 (26.8)	62 (35.2)	
Small artery occlusion	35 (14.8)	58/260 (22.3)		27 (16.1)	20/157 (12.7)		20 (13.4)	35 (19.9)	
Other determined cause	4 (1.7)	5/260 (1.9)		4 (2.4)	1/157 (0.6)		1 (0.7)	0 (0.0)	
Cardioembolic	3 (1.3)	3/260 (1.2)		1 (0.6)	1/157 (0.6)		0 (0.0)	4 (2.3)	
Duration of hospitalization, day	11 (10–12)	11 (10–12)	0.46	11 (10–12)	11 (10–12)	0.62	11 (10–12)	11 (10–12)	0.83

	70 to <75 years			75 to <80 years			≥80 years		
	RIC (*N* = 134)	Control (*N* = 144)	*p* Value	RIC (*N* = 104)	Control (*N* = 102)	*p* Value	RIC (*N* = 71)	Control (*N* = 72)	*p* Value
Age, y	72 (70–73)	72 (71–73)	0.04	77 (76–78)	77 (75–78)	0.36	83 (81–85)	83 (81–85)	0.38
Sex (F)	57 (42.5)	56 (38.9)	0.57	55 (52.9)	48 (47.1)	0.40	36 (50.7)	30 (41.7)	0.28
Current smoker	25/133 (18.8)	22/140 (15.7)	0.64	15/100 (15.0)	17/94 (18.1)	0.27	11/69 (15.9)	9/71 (12.7)	0.48
Current drinker^a^	15/133 (11.3)	13/141 (9.2)	0.56	7/103 (6.8)	12/98 (12.2)	0.21	5/70 (7.1)	1/69 (1.4)	0.24
Hypertension	82/133 (61.7)	85/142 (59.9)	0.76	64/101 (63.4)	57/101 (56.4)	0.32	43/70 (61.4)	45/71 (63.4)	0.81
Diabetes	31/133 (23.3)	35/143 (24.5)	0.82	19 (18.3)	20/101 (19.8)	0.78	11 (15.5)	15 (20.8)	0.41
Previous ischemic or hemorrhagic stroke^b^	53/133 (39.8)	53/143 (37.1)	0.63	34/102 (33.3)	37 (36.3)	0.66	21/70 (30.0)	23 (31.9)	0.80
Previous transient ischemic attack	2 (1.5)	1/143 (0.7)	0.52	1 (1.0)	1 (1.0)	0.99	0 (0.0)	1 (1.4)	0.32
Systolic blood pressure, mmHg	150 (140–164)	150 (140–166)	0.44	157 (140–168)	156 (140–168)	0.67	142 (130–160)	150 (140–176)	0.03^*^
Diastolic blood pressure, mmHg	90 (80–98)	90 (80–95)	0.83	90 (79–94)	85 (79–95)	0.71	82 (75–90)	85 (79–95)	0.16
Fasting blood glucose at randomization, mmol/L	6.40 (5.38–7.99)	6.35 (5.44–7.94)	0.91	6.24 (5.40–7.77)	6.28 (5.46–8.61)	0.72	6.03 (5.36–6.88)	6.00 (5.36–7.86)	0.52
NIHSS score at randomization^c^	7 (6–9)	7 (6–9)	0.97	7 (6–11)	7 (6–9)	0.80	7 (6–11)	6 (6–9)	0.12
Onset‐to‐treatment time, hour	24.4 (11.3–33.1)	26.3 (18.1–35.5)	0.40	20.3 (10.9–30.9)	23.7 (10.2–31.9)	0.56	26.0 (13.5–34.6)	22.0 (10.8–31.0)	0.15
Estimated premorbid function (mRS)^d^									
No symptoms (score, 0)	100 (74.6)	109 (75.7)	0.84	82 (78.8)	73 (71.6)	0.23	47 (66.2)	54 (75.0)	0.25
Symptoms without any disability (score, 1)	34 (25.4)	35 (24.3)		22 (21.2)	29 (28.4)		24 (33.8)	18 (25.0)	
Presumed stroke cause^e^									
Undetermined cause	79/133 (59.4)	72 (50.0)	0.40	46 (44.2)	52 (51.0)	0.45	42 (59.2)	38 (52.8)	0.50
Large artery atherosclerosis	33/133 (24.8)	50 (34.7)		37 (35.6)	32 (31.4)		19 (26.8)	20 (27.8)	
Small artery occlusion	18/133 (13.5)	19 (13.2)		16 (15.4)	16 (15.7)		7 (9.9)	13 (18.1)	
Other determined cause	1/133 (0.8)	1 (0.8)		3 (2.9)	0 (0.0)		1 (1.4)	0 (0.0)	
Cardioembolic	2/133 (1.5)	2 (1.5)		2 (1.9)	2 (2.0)		2 (2.8)	1 (1.4)	
Duration of hospitalization, day	11 (10–12)	11 (10–12)	0.15	10 (9–110	11 (10–12)	0.15	10 (9–12)	11 (10–12)	0.52

*Note*: Data were shown with number (percentage, %) or median (interquartile range).

Abbreviations: mRS, modified Rankin Scale; NIHSS, National Institute of Health Stroke Scale; RIC, remote ischemic conditioning.

* *p* value <0.05.

^a^ Current drinker means consuming alcohol at least once a week within 1 year before onset of the disease and consuming alcohol continuously for more than 1 year.

^b^ Previous ischemic stroke referred only to the patients with pre‐stroke mRS ≤1.

^c^ Patients with NIHSS scores of 6–16 were eligible for this study; NIHSS scores range from 0 to 42, with higher scores indicating more severe neurologic deficit.

^d^ Scores on the modified Rankin Scale (mRS) of functional disability range from 0 (no symptoms) to 6 (death).

^e^ The presumed stroke cause was classified according to the Trial of Org 10,172 in Acute Stroke Treatment (TOAST) classification systemizing clinical findings, brain imaging, and laboratory tests. Other determined causes included pulmonary embolism, peripheral vessel incident, and cardiovascular incident.^17^

**FIGURE 2:**
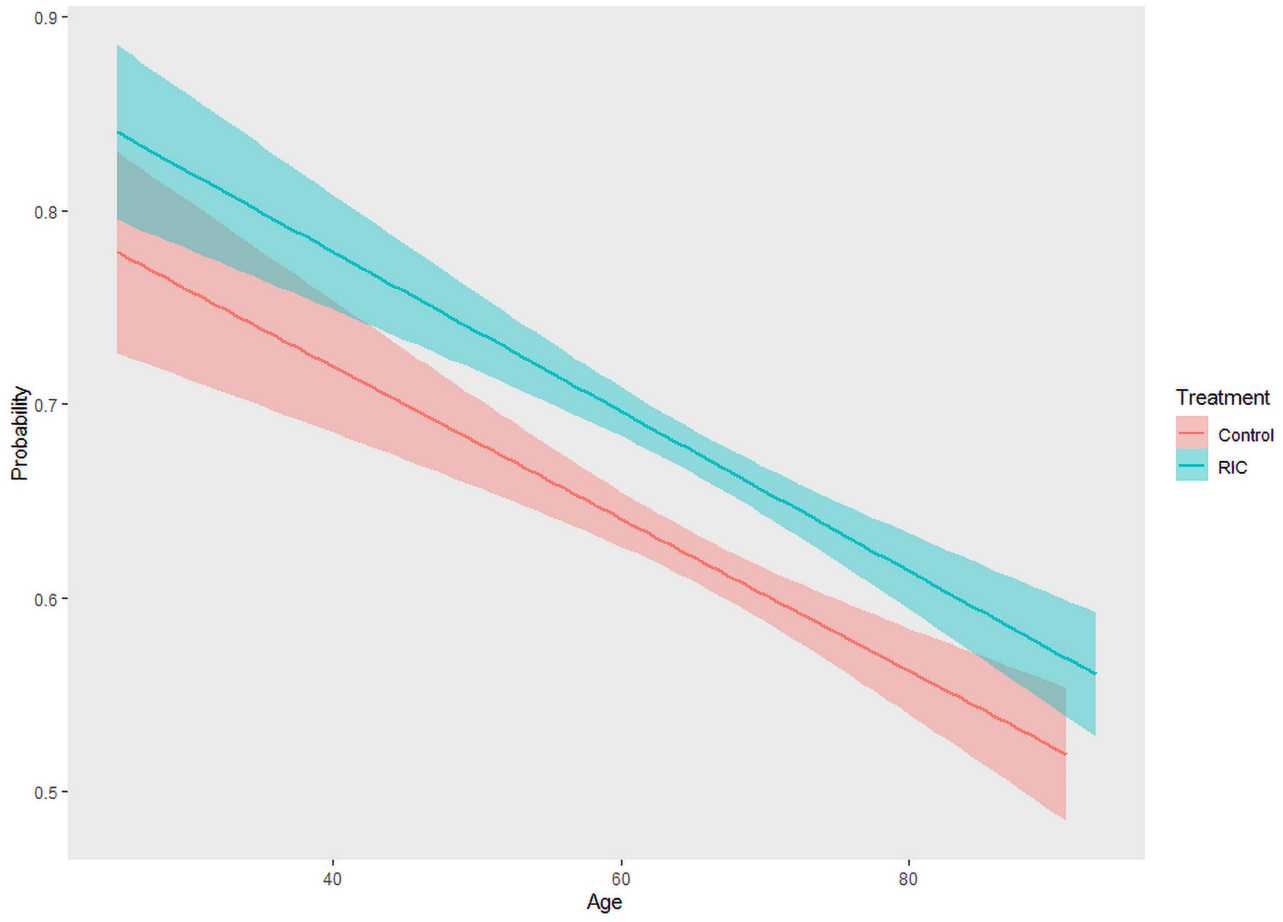
Probability Curves for Excellent Functional Outcome at 90 days Stratified according to Treatment Group. Increasing patients' age was associated with decreasing likelihood of excellent functional outcome at 90 days in RIC group (adjusted OR, 0.98; 95% CI, 0.97–0.99) and control group: (adjusted OR, 0.99; 95% CI, 0.97–1.00). No significant treatment‐by‐age interaction was observed (adjusted *p* = 0.99). The *X*‐axis represents patients' age, and the *Y*‐axis represents the probability of excellent functional outcome at 90 days. The lines represent best‐fit line of probability, and the shaded areas represent their 95% CIs. Excellent functional outcome was defined as mRS scoring 0 to 1 at 90 days after randomization. Control group included patients who received no RIC treatment. CI, confidence intervals; mRS, modified Rankin Scale; OR, odds ratio; RIC, remote ischemic conditioning.

**FIGURE 3:**
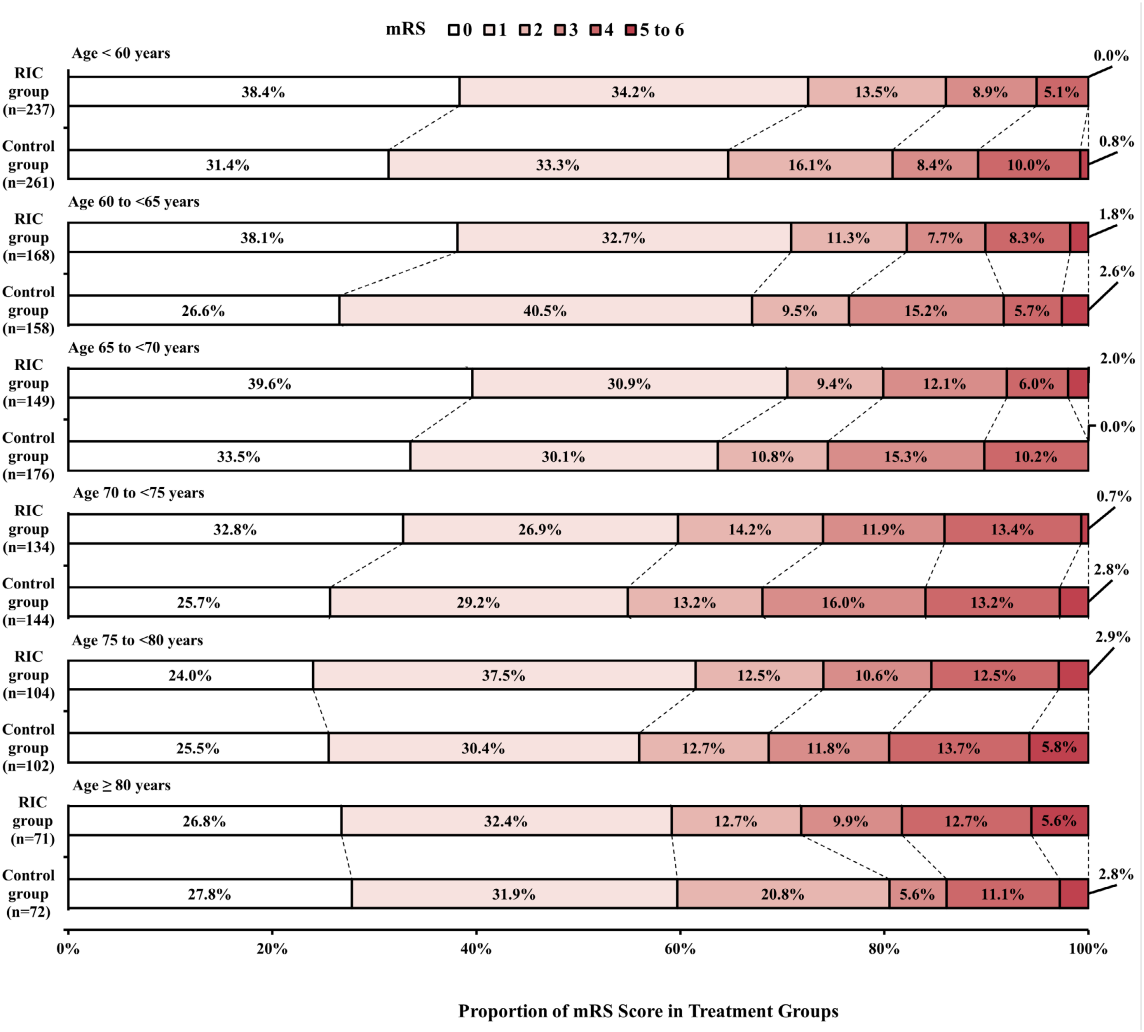
Distribution of 90‐day mRS Score in Treatment Groups Stratified According to Age. Scores on the mRS range from 0 to 6. 0 = no symptoms, 1 = symptoms without clinically significant disability, 2 = slight disability, 3 = moderate disability, 4 = moderately severe disability, 5 = severe disability, and 6 = death. Control group included patients who received no RIC treatment. mRS, modified Rankin Scale; RIC, remote ischemic conditioning.

The probability of mRS score of 0 to 1 in the RIC group was always higher than that in the control group at every age stage, but decreased with increasing age in two groups (Figure 2). The raw distribution of mRS score at 90 days in treatment groups stratified according to patient age is shown in Figure 3. We estimated the association between RIC treatment and functional outcomes in each age group. The primary analyses did not show significantly increased odds in excellent outcome of RIC group in the age <60 years group (adjusted RD, 6.8%; 95% CI, −1.6% to 15.1%; *p* = 0.11), in the age 60 to <65 years group (adjusted RD, 3.1%; 95% CI, −7.2% to 13.3%; *p* = 0.56), in the age 65 to <70 years group (adjusted RD, 3.5%; 95% CI, −6.8% to 13.8%; *p* = 0.51), in the age 70 to <75 years group (adjusted RD, 4.7%; 95% CI, −7.1% to 16.4%; *p* = 0.61), in the age 75 to <80 years group (adjusted RD, 5.7%; 95% CI, −7.8% to 19.1%; *p* = 0.41), and in the age ≥ 80 years group (adjusted RD, −2.6%; 95% CI, −18.8% to 13.5%; *p* = 0.75). Similar results were also obtained in the unadjusted analysis (Table 3). The sensitivity analysis showed that primary outcome did not show significant difference between three age groups, which was consistent with that between six age groups (Table S1).

**TABLE 3:** Outcomes Comparison between Treatment Groups according to Age Groups.

Outcomes	Age	Groups	No. of events (%)	Treatment effect metric	Unadjusted		Adjusted^a^		*p* Value for interaction	
					Treatment difference (95% CI)	*p* Value	Treatment difference (95% CI)	*p* Value	Unadjusted	Adjusted^b^
mRS score of 0 to 1 within 90 days^c^	<60 years	RIC (*N* = 237)	172 (72.6)	RD, %^d^	7.8 (−0.3 to 15.9)	0.06	6.8 (−1.6 to 15.1)	0.11	0.42	0.47
		Control (*N* = 261)	169 (64.8)							
	60 to <65 years	RIC (*N* = 168)	119 (70.7)		3.7 (−6.3 to 13.8)	0.47	3.1 (−7.2 to 13.3)	0.56		
		Control (*N* = 158)	106 (67.1)							
	65 to <70 years	RIC (*N* = 149)	105 (70.5)		6.8 (−3.4 to 17.0)	0.19	3.5 (−6.8 to 13.8)	0.51		
		Control (*N* = 176)	112 (63.6)							
	70 to <75 years	RIC (*N* = 134)	80 (59.7)		4.8 (−6.8 to 16.5)	0.41	4.7 (−7.1 to 16.4)	0.61		
		Control (*N* = 144)	79 (54.9)							
	75 to <80 years	RIC (*N* = 104)	64 (61.5)		5.7 (−7.8 to 19.1)	0.41	5.7 (−7.8 to 19.1)	0.41		
		Control (*N* = 102)	57 (55.9)							
	≥80 years	RIC (*N* = 71)	42 (59.2)		−0.6 (−16.7 to 15.5)	0.95	−2.6 (−18.8 to 13.5)	0.75		
		Control (*N* = 72)	43 (59.7)							
mRS score of 0 to 2 within 90 days^c^	<60 years	RIC (*N* = 237)	202 (85.2)	RD, %^d^	4.4 (−2.2 to 11.0)	0.19	3.5 (−3.2 to 10.3)	0.31	0.31	0.34
		Control (*N* = 261)	211 (80.8)							
	60 to <65 years	RIC (*N* = 168)	138 (82.1)		5.6 (−3.2 to 14.3)	0.22	5.5 (−3.5 to 14.5)	0.23		
		Control (*N* = 158)	121 (76.6)							
	65 to <70 years	RIC (*N* = 149)	119 (79.9)		5.4 (−3.7 to 14.5)	0.24	3.1 (−6.3 to 12.4)	0.52		
		Control (*N* = 176)	131 (74.4)							
	70 to <75 years	RIC (*N* = 134)	99 (73.9)		5.8 (−4.8 to 16.5)	0.28	7.2 (−3.6 to 17.9)	0.19		
		Control (*N* = 144)	98 (68.1)							
	75 to <80 years	RIC (*N* = 104)	77 (74.0)		5.4 (−6.9 to 17.7)	0.39	5.4 (−6.9 to 17.7)	0.39		
		Control (*N* = 102)	70 (68.6)							
	≥80 years	RIC (*N* = 71)	52 (73.2)		−7.3 (−21.1 to 6.5)	0.30	−9.5 (−23.5 to 4.4)	0.18		
		Control (*N* = 72)	58 (80.6)							
mRS distribution within 90 days^c^	<60 years	RIC (*N* = 237)	–	OR^e^	1.43 (1.04 to 1.96)	0.03	1.35 (0.98 to 1.87)	0.07	0.51	0.45
		Control (*N* = 261)	–							
	60 to <65 years	RIC (*N* = 168)	–		1.42 (0.96 to 2.11)	0.08	1.40 (0.94 to 2.09)	0.10		
		Control (*N* = 158)	–							
	65 to <70 years	RIC (*N* = 149)	–		1.32 (0.89 to 1.96)	0.17	1.23 (0.82 to 1.84)	0.31		
		Control (*N* = 176)	–							
	70 to <75 years	RIC (*N* = 134)	–		1.31 (0.86 to 2.00)	0.21	1.31 (0.86 to 2.00)	0.21		
		Control (*N* = 144)	–							
	75 to <80 years	RIC (*N* = 104)	–		1.16 (0.71 to 1.89)	0.56	1.16 (0.71 to 1.89)	0.56		
		Control (*N* = 102)	–							
	≥80 years	RIC (*N* = 71)	–		0.87 (0.48 to 1.57)	0.65	0.79 (0.44 to 1.43)	0.44		
		Control (*N* = 72)	–							

*Note*: Treatment effect is presented as RD or OR with its 95% CI of comparison between groups, analyzed by unadjusted and adjusted analyses.

Abbreviations: CI, confidence intervals; mRS, modified Rankin Scale; OR, odds ratio; RD, risk difference.

^a^ Adjusted for covariates compared between RIC and control group with *p* value <0.1 in each age group (age, current smoker, current drinker, presumed stroke cause in the age <60 years, current drinker, systolic blood pressure, diastolic blood pressure in the age 60 to <65 years, hypertension, and presumed stroke cause in the age 65 to <70 years, age in the age 70 to <75 years, no imbalance in the age 75 to <80 years, and systolic blood pressure in the age ≥80 years).

^b^ Adjusted for covariates compared between age groups with *p* value <0.1 in Table 1.

^c^ mRS scores range from 0 to 6: 0 = no symptoms, 1 = symptoms without clinically significant disability, 2 = slight disability, 3 = moderate disability, 4 = moderately severe disability, 5 = severe disability, and 6 = death.

^d^ Calculated using generalized linear model.

^e^ Calculated using ordinal logistic analysis.

For secondary outcomes, the proportion of patients with mRS of 0–2 at 90 days and mRS distribution at 90 days were not significantly different between RIC and control groups at each age stage (Table 3).

For the interaction between patient age and effect of RIC treatment on primary and secondary outcomes, the results are shown in Table 3. There was no significant interaction for the outcomes when the analyses were performed between age groups.

## 4 DISCUSSION

In this post hoc analysis of RICAMIS, we divided patients with acute moderate ischemic stroke into six groups according to patient age at admission, with the aim to explore the effect of patient age on long‐term functional outcomes after stroke in patients receiving RIC treatment. The results showed that higher proportion of excellent functional outcomes at 90 days were found in the RIC group compared with control group in each age group and decreased with increasing age, but no significant difference between groups and interaction was identified according to patient age.

This is the first study to investigate the effect of patient age on RIC treatment for functional outcomes after acute ischemic stroke. The present study divided patients' age into more groups than that in the prespecified age subgroup of RICAMIS trial,^9^ which further explored the age characteristic of patients who maybe benefit from RIC treatment. In this study, RIC treatment was shown to be superior to usual care alone with respect to likelihood of excellent functional outcome and favorable functional outcome at 90 days, but with no significant difference in each age group. The neutral results of RIC treatment effect may be due to the lower statistical power limited by the insufficient sample size in each group. Subsequently, we investigated the interaction for the outcomes between effect of RIC treatment and patient age, and the superiority of RIC treatment compared with usual care alone was consistent across all age groups.These results were consistent with the subgroup analysis in RICAMIS trial and in RICA (Chronic Remote Ischaemic Conditioning in Patients with Symptomatic Intracranial Atherosclerotic Stenosis) trial through comparing two age groups (<65 years and ≥65 years),^9^, ^10^ both of which showed better efficacy of RIC in patients <65 years versus ≥65 years, but no interaction between RIC treatment effect and age was identified in this study. Furthermore, we found that likelihood of excellent functional outcome gradually decreased as age increased. Previous study showed that RIC treatment improved neurological function after experimental stroke by modulating inflammation,^18^ in which the levels of systemic proinflammatory cytokines increased with advancing age.^19^ Thus, we inferred that patient age may affect functional outcomes following RIC treatment through modulating inflammation. However, the explanation warrants investigation in the future. Additionally, there were more female in patients with higher age and imbalanced onset‐to‐treatment time between age groups. In the preclinical study, more neuroprotective effects of RIC were found in male animals,^20^ while our recent analysis did not find the effect of sex on efficacy of RIC.^21^ As early RIC initiation within 24 h of onset may be associated with better functional outcome,^22^ imbalanced onset‐to‐treatment time between age groups partially confounded the results. Although these imbalanced baseline characteristics may influence effects of RIC between age groups, the primary analyses for interaction through adjusting unbalanced variables between age groups may mitigate the impact of these imbalances. Collectively, these results suggested that efficacy of RIC treatment on long‐term functional outcomes after ischemic stroke may be consistent according to patient age, but decreased with age increasing.

We admitted there are some limitations in this study. First, in this post hoc analysis, patients with age <60 years were slightly less numerous than those with age 60 to <70 years and age ≥70 years. Thus, this analysis was hampered by inadequate statistical power due to an imbalanced as well as relatively smaller sample size in groups, which resulted in comparison with no significant difference. However, we performed sensitivity analysis with three age groups to verify the stability of results in the present study. Second, as RICAMIS trial excluded patients who had received intravenous thrombolysis or endovascular therapy, the findings could not represent the consistency RIC treatment effect according to patient age after receiving reperfusion treatments. Third, the generalizability of the results would need to be validated in other cohorts, particularly in a non‐Chinese population. Finally, we interpret our findings with caution due to the exploratory nature of this post hoc analysis. Thus, these findings warrant confirmation.

## 5 CONCLUSION

This post hoc exploratory analysis of RICAMIS trial suggests that the effect of RIC treatment on long‐term functional outcome at 90 days may be attenuated with increasing age in adults with acute ischemic stroke who are not candidate for intravenous thrombolysis or endovascular therapy. This finding needs to be confirmed in future trials.

## AUTHOR CONTRIBUTIONS

HSC contributed to the conception and design of the study; YC and JZ contributed to acquisition and analysis of data; YC contributed to drafting the text and preparing the figures.

## FUNDING INFORMATION

This study was supported by grants from the Science and Technology Project Plan of Liaoning Province (2022JH2/101500020). The funders of the study had no role in the study design, data collection, data analysis, data interpretation, or writing of the report.

## CONFLICT OF INTEREST STATEMENT

The authors declare that there is no conflict of interests.

## Supporting information


Table S1–S3

## Data Availability

The datasets analyzed during the current study are available from the corresponding author on reasonable request.
