# Supplementary material for: Age and efficacy of remote ischemic conditioning in acute ischemic stroke
Source: CNS Neurosci Ther. 2023 Sep 4;30(3):e14451. doi: 10.1111/cns.14451 (PMC10916442; doi:10.1111/cns.14451)
Supplement: Supplementary file 1 — Table S1–S3 [file CNS-30-e14451-s001.docx]

**Supplementary Materials**

**Table S1 Sensitivity Analysis.**

| **Outcomes** | **Age** | **Groups** | **No. of events (%)** | **Treatment**  **effect**  **metric** | **Unadjusted** | | **Adjusted** † | | ***P* Value for interaction** | |
| --- | --- | --- | --- | --- | --- | --- | --- | --- | --- | --- |
|  |  |  |  |  | **Treatment difference (95% CI)** | ***P* Value** | **Treatment difference (95% CI)** | ***P* Value** | **Unadjusted** | **Adjusted** ‡ |
| mRS score of 0 to 1 within 90 days § | <60 years | RIC (N=237) | 172 (72.6) | RD,% ¶ | 7.8 (-0.3 to 15.9) | 0.06 | 6.8 (-1.6 to 15.1) | 0.11 | 0.50 | 0.54 |
|  |  | Control (N=261) | 169 (64.8) |  |  |  |  |  |  |  |
|  | 60 to < 70 years | RIC (N=317) | 224 (70.7) |  | 5.4 (-1.8 to 12.5) | 0.14 | 3.9 (-3.3 to 11.0) | 0.29 |  |  |
|  |  | Control (N=334) | 218 (65.3) |  |  |  |  |  |  |  |
|  | ≥ 70 years | RIC (N=309) | 186 (60.2) |  | 3.9 (-3.8 to 11.6) | 0.32 | 4.7 (-3.1 to 12.5) | 0.24 |  |  |
|  |  | Control (N=318) | 179 (56.3) |  |  |  |  |  |  |  |

Treatment effect is presented as RD with its 95% CI of comparison between groups, analyzed by unadjusted and adjusted analyses. Abbreviations: CI, confidence intervals; mRS, modified Rankin Scale; RD, risk difference.

† Adjusted for covariates compared between RIC and Control group with *P* value < 0.1 in each age group (age, current smoker, current drinker, presumed stroke cause in the age <60 years, presumed stroke cause in the age 60 to <70 years, duration of hospitalization in the age ≥70 years).

‡ Adjusted for covariates compared between age groups with *P* value <0.1 (age, sex, current smoker, current drinker, diabetes, previous ischemic or hemorrhagic stroke, diastolic blood pressure, onset-to-treatment time, and duration of hospitalization).

§ mRS scores range from 0 to 6: 0 = no symptoms, 1 = symptoms without clinically significant disability, 2 = slight disability, 3 = moderate disability, 4 = moderately severe disability, 5 = severe disability, and 6 = death.

¶ Calculated using generalized linear model.

**Table S2 Baseline Characteristics of Patients in Trichotomized Age Categories.**

|  | **< 60 years**  **(N=498)** | **60 to < 70 years**  **(N=651)** | **≥ 70 years**  **(N=627)** | ***P* Value** |
| --- | --- | --- | --- | --- |
| Age, median (IQR), y | 54 (50-57) | 64 (62-67) | 75 (72-79) | < 0.001 |
| Sex (F), No. (%) | 116 (23.3) | 208 (32.0) | 282 (45.0) | <0.001 |
| Current smoker, No. (%) | 202/484 (41.7) | 204/626 (32.6) | 99/607 (16.3) | < 0.001 |
| Current drinker, No. (%)* | 102/488 (20.9) | 85/633 (13.4) | 53/614 (8.6) | < 0.001 |
| Comorbidities, No. (%)† | | | | |
| Hypertension | 301/492 (61.2) | 406/643 (63.1) | 376/618 (60.8) | 0.67 |
| Diabetes | 124/497 (24.9) | 176/649 (27.1) | 131/624 (21.0) | 0.04 |
| Previous ischemic or hemorrhagic stroke‡ | 120 (24.1) | 228/645 (35.3) | 221/622 (35.5) | < 0.001 |
| Previous transient ischemic attack | 6 (1.2) | 10/648 (1.5) | 6/626 (1.0) | 0.64 |
| Blood pressure at randomization, median (IQR), mmHg | | | | |
| Systolic | 150 (140-163) | 150 (140-165) | 150 (140-167) | 0.55 |
| Diastolic | 90 (82-100) | 90 (80-98) | 88 (80-94) | < 0.001 |
| Blood glucose, median (IQR), mmol/L | 6.29 (5.36-8.91) | 6.35 (5.44-8.44) | 6.21 (5.40-7.92) | 0.29 |
| Baseline NIHSS score, median (IQR)§ | 7 (6-9) | 7 (6-8) | 7 (6-9) | 0.10 |
| Estimated premorbid function (mRS), No. (%)\|\| | | | | |
| No symptoms (score, 0) | 380 (76.3) | 487 (74.8) | 465 (74.2) | 0.71 |
| Symptoms without any disability (score, 1) | 118 (23.7) | 164 (25.2) | 162 (25.8) |  |
| OTT, median (IQR), h | 26.2 (13.0-37.0) | 25.5 (13.8-35.0) | 24.3 (12.0-33.2) | 0.04 |
| Duration of hospitalization, median (IQR), d | 11 (10-12) | 11 (10-12) | 10 (10-12) | 0.07 |
| Presumed stroke cause, No. (%)¶ | | | | |
| Undetermined cause | 257/497 (51.7) | 343/650 (52.8) | 329/626 (52.6) | 0.40 |
| Large artery atherosclerosis | 132/497 (26.6) | 193/650 (29.7) | 191/626 (30.5) |  |
| Small artery occlusion | 93/497 (18.7) | 102/650 (15.7) | 89/626 (14.2) |  |
| Other determined cause | 9/497 (1.8) | 6/650 (0.9) | 7/626 (1.1) |  |
| Cardioembolic | 6/497 (1.2) | 6/650 (0.9) | 10/626 (1.6) |  |

Abbreviations: IQR, interquartile range; OTT, time from onset of symptom to remote ischemic conditioning treatment; NIHSS, National Institute of Health Stroke Scale; mRS, modified Rankin Scale.

* Current drinker means consuming alcohol at least once a week within 1 year before onset of the disease and consuming alcohol continuously for more than 1 year.

† The comorbidities were based on the patient or family report.

‡ Previous ischemic stroke referred only to the patients with pre-stroke mRS ≤ 1.

§ Patients with NIHSS scores of 6 to 16 were eligible for this study; NIHSS scores range from 0 to 42, with higher scores indicating more severe neurologic deficit.

|| Scores on the modified Rankin Scale (mRS) of functional disability range from 0 (no symptoms) to 6 (death).

¶ The presumed stroke cause was classified according to the Trial of Org 10172 in Acute Stroke Treatment (TOAST) classification system using clinical findings, brain imaging, and laboratory tests. Other determined causes included pulmonary embolism, peripheral vessel incident, and cardiovascular incident.

**Table S3 Baseline Characteristics of Patients between RIC and Control Groups in Each Age Category.**

|  | **< 60 years** | | | **60 to < 70 years** | | | **≥ 70 years** | | |
| --- | --- | --- | --- | --- | --- | --- | --- | --- | --- |
|  | **RIC (N=237)** | **Control (N=261)** | **P Value** | **RIC (N=317)** | **Control (N=334)** | **P Value** | **RIC (N=309)** | **Control (N=318)** | **P Value** |
| Age, median (IQR), y | 55 (50-57) | 54 (50-56) | 0.07 | 64 (62-67) | 65 (62-67) | 0.12 | 75 (72-79) | 75 (73-79) | 0.99 |
| Sex (F), No. (%) | 51 (21.5) | 65 (24.9) | 0.37 | 108 (34.1) | 100 (29.9) | 0.26 | 148 (47.9) | 134 (42.1) | 0.15 |
| Current smoker, No. (%) | 104/231 (45.0) | 98/253 (38.7) | 0.03 | 104/306 (34.0) | 100/320 (31.3) | 0.39 | 51/302 (16.9) | 48/305 (15.7) | 0.25 |
| Current drinker, No. (%)* | 59/233 (25.3) | 43/255 (16.9) | 0.06 | 51/309 (16.5) | 34/324 (10.5) | 0.15 | 27/306 (8.8) | 26/308 (8.4) | 0.15 |
| Comorbidities, No. (%)† | | | | | | | | | |
| Hypertension | 142/235 (60.4) | 159/257 (61.9) | 0.74 | 200/313 (63.9) | 206/330 (62.4) | 0.70 | 189/304 (62.2) | 187/314 (59.6) | 0.51 |
| Diabetes | 61 (25.7) | 63/260 (24.2) | 0.70 | 86 (27.1) | 90/332 (27.1) | 0.99 | 61/308 (19.8) | 70/316 (22.2) | 0.47 |
| Previous ischemic or hemorrhagic stroke‡ | 56 (23.6) | 64 (24.5) | 0.82 | 116/316 (36.7) | 112/329 (34.0) | 0.48 | 108/305 (35.4) | 113/317 (35.6) | 0.95 |
| Previous transient ischemic attack | 3 (1.3) | 3 (1.1) | 0.91 | 5/315 (1.6) | 5/333 (1.5) | 0.93 | 3 (1.0) | 3/317 (0.9) | 0.98 |
| Blood pressure at randomization, median (IQR), mmHg | | | | | | | | | |
| Systolic | 150 (140-164) | 150 (140-163) | 0.59 | 150 (140-162) | 150 (140-165) | 0.83 | 150 (140-165) | 150 (140-170) | 0.16 |
| Diastolic | 90 (84-100) | 90 (80-100) | 0.76 | 90 (80-97) | 90 (80-98) | 0.62 | 88 (79-93) | 88 (80-95) | 0.45 |
| Blood glucose, median (IQR), mmol/L | 6.45 (5.40-8.91) | 6.20 (5.30-8.95) | 0.35 | 6.24 (5.6-8.61) | 6.40 (5.44-8.31) | 0.84 | 6.20 (5.40-7.70) | 6.23 (5.41-8.09) | 0.56 |
| Baseline NIHSS score, median (IQR)§ | 7 (6-9) | 7 (6-9) | 0.62 | 7 (6-9) | 7 (6-8) | 0.31 | 7 (6-10) | 7 (6-9) | 0.38 |
| Estimated premorbid function (mRS), No. (%)\|\| | | | | | | | | | |
| No symptoms (score, 0) | 184 (77.6) | 196 (75.1) | 0.51 | 234 (73.8) | 253 (75.7) | 0.57 | 229 (74.1) | 236 (74.2) | 0.98 |
| Symptoms without any disability (score, 1) | 53 (22.4) | 65 (24.9) |  | 83 (26.2) | 81 (24.3) |  | 80 (25.9) | 82 (25.8) |  |
| OTT, median (IQR), h | 27.0 (12.6-38.0) | 25.9 (13.9-35.2) | 0.42 | 26.5 (14.6-36.1) | 25.0 (12.6-34.3) | 0.22 | 23.8 (11.4-33.1) | 24.5 (12.3-34.2) | 0.39 |
| Duration of hospitalization, median (IQR), d | 11 (10-12) | 11 (10-12) | 0.46 | 11 (10-12) | 11 (10-12) | 0.93 | 10 (10-12) | 11 (10-12) | 0.02 |
| Presumed stroke cause, No. (%)¶ | | | | | | | | | |
| Undetermined cause | 138 (58.2) | 119/260 (45.8) | 0.07 | 181 (57.1) | 162/333 (48.6) | 0.03 | 167/308 (54.2) | 162 (50.9) | 0.55 |
| Large artery atherosclerosis | 57 (24.1) | 75/260 (28.8) |  | 83 (26.2) | 110/333 (33.0) |  | 89/308 (28.9) | 102 (32.1) |  |
| Small artery occlusion | 35 (14.8) | 58/260 (22.3) |  | 47 (14.8) | 55/333 (16.5) |  | 41/308 (13.3) | 48 (15.1) |  |
| Other determined cause | 4 (1.7) | 5/260 (1.9) |  | 5 (1.6) | 1/333 (0.3) |  | 5/308 (1.6) | 2 (0.6) |  |
| Cardioembolic | 3 (1.3) | 3/260 (1.2) |  | 1 (0.3) | 5/333 (1.5) |  | 6/308 (1.9) | 4 (1.3) |  |

Abbreviations: IQR, interquartile range; OTT, time from onset of symptom to remote ischemic conditioning treatment; NIHSS, National Institute of Health Stroke Scale; mRS, modified Rankin Scale; RIC, remote ischemic conditioning.

* Current drinker means consuming alcohol at least once a week within 1 year before onset of the disease and consuming alcohol continuously for more than 1 year.

† The comorbidities were based on the patient or family report.

‡ Previous ischemic stroke referred only to the patients with pre-stroke mRS ≤ 1.

§ Patients with NIHSS scores of 6 to 16 were eligible for this study; NIHSS scores range from 0 to 42, with higher scores indicating more severe neurologic deficit.

|| Scores on the modified Rankin Scale (mRS) of functional disability range from 0 (no symptoms) to 6 (death).

¶ The presumed stroke cause was classified according to the Trial of Org 10172 in Acute Stroke Treatment (TOAST) classification system using clinical findings, brain imaging, and laboratory tests. Other determined causes included pulmonary embolism, peripheral vessel incident, and cardiovascular incident.
